# *Notes from the Field:* Investigation of Unapproved Botulinum Toxin Product Administered at a Medical Spa — Colorado, 2025–2026

**DOI:** 10.15585/mmwr.mm7539a2

**Published:** 2026-10-08

**Authors:** Pallavi Oruganti, Natalia G. Riemer, Cayla E. Mayle, Nicholas R. Laramee, Kerri B. Brown, Janell L. Bezdek, Christopher A. Czaja, Ingrid R. Hewitson, Shireen Banerji, Liz Lamere, Ethel V. Taylor, Sarah Venti, Kristle Green, Rachel H. Jervis

**Affiliations:** ^1^Epidemic Intelligence Service, CDC; ^2^Colorado Department of Public Health and Environment; ^3^Boulder County Public Health, Boulder, Colorado; ^4^Rocky Mountain Poison & Drug Safety, Denver, Colorado; ^5^Division of Foodborne, Waterborne, and Environmental Diseases, National Center for Emerging and Zoonotic Infectious Diseases, CDC; ^6^Office of Compliance, Center for Drug Evaluation and Research, Food and Drug Administration, Silver Spring, Maryland.

**Summary**

**What is already known about this topic?**

Use of counterfeit or unapproved botulinum neurotoxin (BoNT) can result in serious illness.

**What is added by this report?**

In 2026, a Colorado poison center report identified use of a BoNT product not approved by the Food and Drug Administration (FDA) at a medical spa; subsequent public health investigation revealed patient safety issues including adverse effects after administration of the unapproved product.

**What are the implications for public health practice?**

Persons should be encouraged to seek BoNT treatment only from licensed providers administering FDA-approved products. Public health investigations of reports of posttreatment adverse events are important to ensure compliance with regulations and patient safety.

In 2002, the Food and Drug Administration (FDA) approved the use of cosmetic botulinum neurotoxin (BoNT) to treat medical and aesthetic conditions, including facial wrinkles (*1*). In 2024, 9.8 million persons received cosmetic BoNT injections in the United States. Adverse events have been documented after injection of BoNT that has been improperly procured, injected by a person without the proper medical credentials, or that has not been approved by FDA for use in the United States (*2*–*4*). During January 2026, Rocky Mountain Poison and Drug Safety (RMPDS) alerted public health authorities of a woman (patient A) reporting symptoms consistent with BoNT adverse effects and illness; her symptoms began 5 days earlier, on the same day she received a BoNT injection from a licensed medical provider at a medical spa.^†^ Patient A reported seeking medical care for these adverse effects on the same day that she reported her symptoms to RMPDS, at which time her primary care provider determined treatment was not recommended. Patient A’s signs and symptoms had improved by the time she contacted RMPDS, and she did not require hospitalization. The Colorado Department of Public Health and Environment (CDPHE), in collaboration with CDC, FDA, and the local public health agency, launched an investigation.

## Investigation and Outcomes

### Identification of Symptomatic Patients

CDPHE and the local public health agency visited the medical spa to evaluate injection safety practices, identify BoNT products administered, and collect product samples for CDC testing. CDPHE sent a text message survey to 52 medical spa patients identified by the provider as having received BoNT at the spa during December 2025–January 2026 to inquire about treatment details and posttreatment signs and symptoms^§^; 27 (47%) patients responded, including two additional symptomatic patients (patients B and C), who were interviewed by telephone. This activity was reviewed by CDC, deemed not research, and conducted consistent with applicable federal law and CDC policy.^¶^

### Receipt of Non–FDA-Approved BoNT Products by Symptomatic Patients

Patients A, B, and C verified receiving an injection of the same non–FDA-approved product by naming the product they received; however, none of the three had been aware that the product was not FDA approved. None of the patients required hospitalization. Patient A sought medical care and reported improvement by the time of the interview (Table). Patients B and C reported similar symptoms that had resolved without medical attention. All three patients were women who received injections from the same lot of a non–FDA-approved BoNT product in the upper brow, glabella, or upper lip ≥1 day of each other and reported symptoms of numbness and tingling, muscle weakness, dysphonia, blurred vision, or neck stiffness on the day of treatment. Although approved in other countries, the administered product is not approved for use in the United States. The BoNT that the medical spa provider reported administering to these symptomatic patients was verified by the manufacturer, confirming that the lot number corresponded to their product records. The product with the lot number administered to the affected patients was not available for testing. CDC testing of a different lot number of the non–FDA-approved product obtained from the medical spa did identify the presence of active BoNT type A but did not identify elevated levels of botulinum toxins or contaminants. 

**TABLE:** Characteristics of symptomatic patients who received unapproved botulinum neurotoxin injections — Colorado, 2025–2026

Characteristic	Patient A	Patient B	Patient C
Sex	Female	Female	Female
Injection site	Upper brow and glabella	Upper brow and upper lip	Upper brow and glabella
Injection dose (total)*	24 units	3 units	10 units
Time from injection to symptom onset	<24 hrs	<24 hrs	<24 hrs
Signs and symptoms	Numbness and tingling in the arms and legs, neck stiffness, dysphonia, headache, and blurred vision	Dysphonia, headache, and blurred vision	Dyspnea, headache, and leg and arm muscle weakness
Outcome	Recovered; sought outpatient health care	Recovered; did not seek health care	Recovered; did not seek health care

* Information regarding the doses administered was provided by the medical spa provider from clinical notes. Botulinum neurotoxin products are priced and purchased by unit; differences in the number of units administered might reflect a patient request for the amount to be administered, promotional offers from the medical spa, or administration during multiple appointments. Total dosage during multiple appointments is displayed.

### Medical Spa Assessment Findings

Both FDA-approved and non–FDA-approved products were found on-site at the medical spa. The medical spa provider reported that non–FDA-approved products were purchased through an online supplier. Other facility assessment findings included inappropriate use of a preservative-containing diluent, use of single-dose vials for multiple patients, inadequate temperature monitoring, and storage and use of reconstituted and unlabeled product past the beyond-use date** (*1*). The provider received a public health order to immediately stop using the unapproved products, and a formal complaint against the provider’s license was submitted to the Colorado Department of Regulatory Agencies. After initiation of the public health investigation, the provider reported ceasing use of the unapproved products.

## Preliminary Conclusions and Actions

Three persons experienced transient neurologic symptoms after injections of an unapproved BoNT product by a licensed provider in Colorado. The clinical features reported included both adverse reactions listed on the package insert of the product and reactions that were not listed on the package insert. Based on findings from the site investigation, potential causes include unknown product concentration, product contamination, improper preparation, and improper administration or storage of the product (*1*). This response highlights the importance of communication and collaboration among poison centers, health care providers, and public health authorities in reporting patients with symptoms after receiving BoNT injections (FDA | MedWatch Online Voluntary Reporting Form 3500). Follow-up public health investigations are important to ensuring regulatory compliance and patient safety. In addition to being subject to other federal laws and regulations, medical spas might be regulated as dispensers under the Drug Supply Chain Security Act (DSCSA).^††^ On April 1, 2026, FDA issued a warning letter to a medical spa in Texas for obtaining botulinum toxin product in violation of DSCSA. In Colorado, administering non–FDA-approved products can be grounds for disciplinary action against a licensed provider by the Department of Regulatory Agencies, the state’s agency responsible for licensing professionals.

BoNT purchased from an unauthorized supplier might not be FDA approved and might be counterfeit, contaminated, improperly stored, or otherwise unsafe (*5*). Persons should be encouraged to seek treatment only from licensed providers administering FDA-approved BoNT products. Providers should not purchase or administer unapproved BoNT products from unauthorized sources.

## Footnotes

† In Colorado, a medical spa is a facility or medical practice providing cosmetic, aesthetic, wellness, longevity, or lifestyle treatments involving the administration or use of prescription drugs, including injectable and sterile drug products.

§ Patients were asked about the following posttreatment signs and symptoms: pain, swelling, or bruising at the injection site; headache or influenza-like symptoms; droopy eyelids or crooked eyebrows; crooked smile or drooling; watery or dry eyes; infection at the injection site; muscle weakness; vision problems (including blurry or double vision); severe fatigue; difficulty talking or swallowing; difficulty breathing; allergic reaction; increased heart rate or chest pain; feelings of pins and needles, numbness, or tingling; and loss of bladder control. LETYBO (letibotulinumtoxinA-wlbg) Label | FDA, BOTOX (onabotulinumtoxinA) Label | FDA

¶ 45 C.F.R. part 46, 21 C.F.R. part 56; 42 U.S.C. Sect. 241(d); 5 U.S.C. Sect. 552a; 44 U.S.C. Sect. 3501 et seq.

** Investigations of adverse events after BoNT administration have noted numerous deviations from FDA recommendations for drug dose, dilution, handling, injection site, and storage.

†† As defined by DSCSA, dispensers are persons who are authorized to dispense or administer prescription drugs to humans.
